# Risk perception of COVID-19 among sub-Sahara Africans: a web-based comparative survey of local and diaspora residents

**DOI:** 10.1186/s12889-021-11600-3

**Published:** 2021-08-18

**Authors:** Emmanuel Kwasi Abu, Richard Oloruntoba, Uchechukwu Levi Osuagwu, Dipesh Bhattarai, Chundung Asabe Miner, Piwuna Christopher Goson, Raymond Langsi, Obinna Nwaeze, Timothy G. Chikasirimobi, Godwin O. Ovenseri-Ogbomo, Bernadine N. Ekpenyong, Deborah Donald Charwe, Khathutshelo Percy Mashige, Tanko Ishaya, Kingsley Emwinyore Agho

**Affiliations:** 1 Department of Optometry and Vision Science, School of Allied Health Sciences, University of Cape Coast, 00233 Cape Coast, Ghana; 2 School of Management and Marketing, Faculty of Business and Law, Curtin University, Bentley, WA 6151 Australia; 3 Translational Health Research Institute (THRI), School of Medicine, Western Sydney University, Campbelltown, NSW 2560 Australia; 4 Discipline of Optometry, School of Health Sciences, African Vision Research Institute (AVRI), University of KwaZulu-Natal, Westville Campus, Durban, 3629 South Africa; 5 Faculty of Health, School of Medicine, Deakin University, Geelong, Victoria Australia; 6 Department of Community Medicine, College of Health Sciences, University of Jos, Jos, Nigeria; 7 Department of Psychiatry, College of Health Sciences, University of Jos, Jos, Nigeria; 8 Health Division, University of Bamenda Bambili, Bamenda, Cameroon; 9 County Durham and Darlington National Health Service (NHS) Foundation, Darlington, DL3 0PD UK; 10 Department of Optometry and Vision Sciences, School of Public Health, Biomedical Sciences and Technology, Masinde Muliro University of Science and Technology, Kakamega, 50100 Kenya; 11 Department of Optometry, Center for Health Sciences, University of Highlands and Islands, Old Perth Road, IV2 3JH Scotland; 12 Department of Optometry, Faculty of Life Sciences, University of Benin, Benin City, Nigeria; 13 Department of Public Health, Faculty of Allied Medical Sciences, College of Medical Sciences, University of Calabar, Calabar, Cross River State Nigeria; 14 Tanzania Food and Nutrition Center, P. O. Box 977, Dar-es Salaam, Tanzania; 15 Department of Computer Science, University of Jos, Jos, Nigeria; 16 School of Health Sciences, Western Sydney University, Campbelltown, NSW 2560 Australia

**Keywords:** Africa, Pandemic, Diaspora, Lockdown, Risk perception, Sub-Sahara Africa, Knowledge, COVID-19

## Abstract

**Background:**

Perceived risk towards the coronavirus pandemic is key to improved compliance with public health measures to reduce the infection rates. This study investigated how Sub-Saharan Africans (SSA) living in their respective countries and those in the diaspora perceive their risk of getting infected by the COVID-19 virus as well as the associated factors.

**Methods:**

A web-based cross-sectional survey on 1969 participants aged 18 years and above (55.1% male) was conducted between April 27th and May 17th 2020, corresponding to the mandatory lockdown in most SSA countries. The dependent variable was the perception of risk for contracting COVID-19 scores. Independent variables included demographic characteristics, and COVID-19 related knowledge and attitude scores. Univariate and multiple linear regression analyses identified the factors associated with risk perception towards COVID-19.

**Results:**

Among the respondents, majority were living in SSA (*n* = 1855, 92.8%) and 143 (7.2%) in the diaspora. There was no significant difference in the mean risk perception scores between the two groups (*p* = 0.117), however, those aged 18–28 years had lower risk perception scores (*p* = 0.003) than the older respondents, while those who were employed (*p* = 0.040) and had higher levels of education (*p* < 0.001) had significantly higher risk perception scores than other respondents. After adjusting for covariates, multivariable analyses revealed that SSA residents aged 39–48 years (adjusted coefficient, β = 0.06, 95% CI [0.01, 1.19]) and health care sector workers (β = 0.61, 95% CI [0.09, 1.14]) reported a higher perceived risk of COVID-19. Knowledge and attitude scores increased as perceived risk for COVID-19 increased for both SSAs in Africa (β = 1.19, 95% CI [1.05, 1.34] for knowledge; β = 0.63, 95% CI [0.58, 0.69] for attitude) and in Diaspora (β = 1.97, 95% CI [1.16, 2.41] for knowledge; β = 0.30, 95% CI [0.02, 0.58] for attitude).

**Conclusions:**

There is a need to promote preventive measures focusing on increasing people’s knowledge about COVID-19 and encouraging positive attitudes towards the mitigation measures such as vaccines and education. Such interventions should target the younger population, less educated and non-healthcare workers.

**Supplementary Information:**

The online version contains supplementary material available at 10.1186/s12889-021-11600-3.

## Introduction

Risk perception refers to people’s subjective assessments of the possibility of outcomes that may follow undesirable events such as disasters and pandemics [1]. The ongoing novel *coronavirus* SARS-CoV2 (COVID-19) pandemic has caused enormous global mortality and public health devastation [2]. While the 2014 West African Ebola Virus Disease (EVD) pandemic was limited to African countries, and the severe acute respiratory syndrome (SARS) of 2002–03 limited to Asian countries, COVID-19 has been a global and unprecedented ‘black swan’ event [3, 4]. COVID-19 infection is highly contagious, and mortality caused by the virus has exceeded 3.4 million deaths as of 27th of May 2021 ─ more than any of its predecessors [5]. It is, therefore no surprise that countries are in a race towards developing and administering an effective vaccine [6, 7].

In response to the COVID-19 global threat [8], the World Health Organization (WHO) immediately raised awareness of healthcare workers around the world [9]. The WHO has also raised funds globally and developed Strategic Preparedness and Response Plans (SPRP) to support and protect poorer countries with weak healthcare systems [10]. The goal of the SPRP was to control infection, limit transmission, communicate key information, provide early acute care, and minimize disastrous economic and social effects. National governments locked down their populations, stopped the mobility of goods and services, closed all schools and universities, and shut all state and international borders with many employees working from homes [11–14]. Nonetheless, these mitigating measures’ success depends upon the public’s readiness to comply, which in turn is inspired by their risk perceptions about the pandemic [15].

Globally, devastating pandemics such as COVID-19 can provide valuable opportunities to learn about human risk perception and attendant behavior [16, 17] and how findings from such studies can be used to inform the allocation of resources within such countries and within international multilateral organizations and agencies such as the WHO [18, 19]. Such studies can also provide an evidence base for the formulation of public health and risk policies. Severe outcomes from natural disasters are often influenced by the level and distribution of economic resources and income within the population of a country (or region) [20, 21]. Several seminal bodies of literature highlight the role of resources or the lack of them in societal responses to disasters [22] and show how positive psychology can contribute to community development during disasters [23]. Culture and risk perception are closely linked and cultural beliefs and values may contribute to the success or otherwise of efforts to control the COVID 19 pandemic [24, 25]. As a result of the different cultural exposures of African residents and Africans living in the diaspora (living outside Africa), this comparative analysis will bring to the fore what specific local context risk management strategies should be implemented by SSA governments. For instance, Quinn et al. showed that people’s attachment to their place of residence affected their perceived disaster-related risks [26]. The findings of this web-based cross-sectional study will highlight the implications of the analysis for what we might expect of Africans living in Africa and Africans living outside Africa as well as policy implications in disaster risk management in general. For policymakers tasked with communicating risk, this research would provide a particularly valuable lens through which we can address the emotional underpinnings of adaptation behavior.

## Methods

### Design and setting of the study

This was an online survey created in Survey monkey to assess the risk perceptions of Africans. The study was conducted between April 27th and May 17th 2020 corresponding to the mandatory lockdown period in most SSA countries. The survey instrument shown in the Supplementary Table, was adapted and developed from the WHO recommended questions [27] and have been used in previous studies [27]. It was not feasible to undertake a conventional Africa-wide community-based sampling survey at this particular period of lockdown and restricted mobility. A one-page project information statement, which doubled as a recruitment poster, was posted/reposted to WhatsApp and Facebook chat groups and individual accounts together with an e-Link to the online survey. The information sheet and poster contained a brief introduction on the background of the study, its objectives, procedures, the voluntary nature of participation, the declaration of anonymity, privacy and confidentiality, as well as instructions for completing the questionnaire.

We also posted the poster and questionnaire on various websites and official accounts of several local organisations and individuals. Survey questionnaires were also sent out by email to selected groups and individuals in all the target countries, relying on the authors’ networks with collaborating academics and local people.

### Questionnaire

The questionnaire was divided into three sections, including demographics, knowledge, risk perception, feeling about self-isolation, attitude towards public health practices to mitigate the spread of COVID-19 (compliance) as presented in Table 1. Most of the items on the questionnaire that assessed the respondent’s knowledge of COVID- 19, required mostly a ‘true’ or ‘false’ or a ‘yes’ or ‘no’ response with an additional “Not sure” option. Each question used a binary scale, and a correct answer was assigned 1 point, whereas an incorrect/unsure answer was assigned 0 points. The knowledge score ranged from 0 to 18 points. These items have been validated elsewhere to have an acceptable internal consistency [28]. To reduce unintended bias, we conducted a statistical test using Kuder Richardson correlation coefficient for binary outcomes by creating two dummy variables. One of the dummy variables included ‘Yes’ and ‘Not sure’ and the other dummy variable was the combination of ‘No’ and ‘Unsure’ and the alpha coefficient for the two dummy variables was 0.86, indicating a strong relationship.

**Table 1:** Survey items for knowledge, attitude and perception towards COVID-19

**Knowledge**	
K1	Are you aware of the Coronavirus disease (COVID-19) outbreak?
K2	Are you aware of the origin of the Coronavirus disease (COVID-19) outbreak?
K3	Do you think Coronavirus disease (COVID-19) outbreak is dangerous?
K5	Do you think Hand Hygiene / Hand cleaning is important to control the spread of the Coronavirus disease (COVID-19) outbreak?
K6	Do you think ordinary residents can wear general medical masks to prevent the infection by the COVID-19 virus?
K7	Do you think there are any specific medicines to treat Coronavirus disease (COVID-19)?
K8	The main clinical symptoms of Coronavirus disease (COVID-19) are: Fever, Fatigue, dry cough, sore throat
K9	Unlike the common cold, stuffy nose, runny nose, and sneezing are less common in persons infected with the COVID-19 virus.
K10	There currently is no effective cure for COVID-2019, but early symptomatic and supportive treatment can help most patients recover from the infection
K11	It is not necessary for children and young adults to take measures to prevent the infection by the COVID-19 virus
K12	COVID-19 individuals cannot spread the virus to anyone if there’s no fever
K13	The COVID-19 virus spreads via respiratory droplets of infected individuals
K14	To prevent getting infected by Coronavirus disease (COVID-19), individuals should avoid going to crowded places such as train stations, religious gatherings, and avoid taking public transportation
K15	Isolation and treatment of people who are infected with the Coronavirus disease (COVID-19) virus are effective ways to reduce the spread of the virus. The observation period is usually 14 days
K16	Not all persons with COVID-2019 will develop to severe cases. Only those who are elderly, have chronic illnesses, and are obese are more likely to be severe cases.
**Risk Perception**	
*Please rate your chances of personal risk of infection with COVID-19 for each of the following?*	
P1	Risk of becoming infected.
P2	Risk of becoming severely infected
P3	Risk of dying from the infection
P4	How much worried are you because of COVID-19?
P5	How likely do you think Coronavirus disease (COVID-19) will continue in your country?
P6	If Coronavirus disease (COVID-19) continues in your country, how concerned would you be that you or your family would be directly affected?
*How do you feel about the Self-isolation?*	
P7	I am worried about self-isolation.
P8	I am bored by self-isolation.
P9	I am frustrated by self-isolation
P10	I am angry because of self-isolation.
P11	I am anxious about self-isolation.
P12	I am angry because of the quarantine.
**Attitude towards public health practices to mitigate the spread of COVID-19 (Compliance)**	
A1	Are you currently or have you been in (domestic/home) quarantine because of COVID-19?
A2	Are you currently or have you been in self-isolation because of COVID-19?
A3	In recent days, have you gone to any crowded place including religious events?
A4	In recent days, have you worn a mask when leaving home?
A5	In recent days, have you been washing your hands with soap and running water for at least 20 s each time?
A6	Since the government gave the directives on preventing getting infected, have you procured your mask and possibly sanitizer?
A7	Have you travelled outside your home in recent days using the public transport
A8	Are you encouraging others that you meet to observe the basic prevention strategies suggested by the authorities?

See Supplementary Table for the full survey item with the response options

For the risk perception items shown in P1 − P6 of Table 1, each question used a Likert scale with five levels, and the scores ranged from 1 for ‘lowest’ and 5 for ‘highest’ with a maximum score range of 5 to 30 points. We determined the Cronbach’s alpha coefficients of the perception items to be 0.84, which indicated a satisfactory internal consistency of perception items. Questions were asked on “How the respondents felt about self-isolation” (P7 − P12) were classified as “Yes” or “No” The Kuder Richardson Cronbach’s alpha coefficient of the “How the respondents felt about the quarantine items” was 0.74, which indicated an acceptable internal consistency. Respondents were also asked about their attitude towards the public health measures put in place by the respective governments to reduce the spread of the virus in items A1-A8. The Likert scale in items A3-A5 was scored as 0 for ‘lowest’ and 4 for “highest” with the score ranging from 0 to 17 points and the alpha coefficients of the attitude items were 0.73 and demonstrated that the internal consistency of the attitude items was satisfactory.

### Characteristics of the participants

Participants were those living in South Africa, Nigeria, Ghana, Kenya, Tanzania and Malawi. Respondents in the diaspora, including those living in the UK, USA, Australia, Canada, New Zealand and Germany, were also included. Recipients were further encouraged to send on or ‘snowball’ the survey questionnaire to other WhatsApp groups and friends that they knew. Eligibility criteria included that respondents had to be of African nationality, aged 18 years or older, able to understand the contents of the poster/questionnaire, and agreed to participate in the study.

### Dependent variable

The dependent variable for this study was the perception of risk for contracting COVID-19, which was categorized as continuous. The items utilized to measure the risk perception of COVID-19 are shown in Table 1 (P1-P6). The responses included very high, high, low, very low, and unlikely. The items ranged from 1 (unlikely) to 5 (very high).

### Independent variables

These included demographic A) characteristics of the participants, which consists of age, gender, marital status, education, employment status, occupation (if employed), religion, if they lived alone, the number of people living together in the household and place of current residence. B), Knowledge about COVID-19 origin, symptoms and prevention. C), Feeling about the practice of self-isolation during COVID-19 lockdown. D) Attitude towards COVID-19 mitigation measures that included the practice of self-isolation, home quarantine (A1 and A2) as well as compliance questions (A3-A8)(see Table 1).

### Sample size determination

The survey assumed a proportion of 50% with 95% confidence and 2.5% margin of error based on a previous study [29]. This is because the main objective of this research was on COVID-19, and there were no previous studies from SSA that examined factors associated with risk perception of 2019-nCoV. An online sample size calculator was used, and we assumed a sample size of approximately 1921, including 20% non-response rate.

#### Statistical analysis

Scores for risk perception were calculated for each of the independent variables and treated as a continuous variable with mean (±standard deviation) risk scores. The risk perception scores ranged from 1 to 30. Risk scores by independent variables were summarized using a t-test for two categorical groups and a one-way analysis of variance (ANOVA) for more than two categorical groups. Univariate linear regression analyses were conducted to assess the unadjusted coefficients (B) with 95% confidence intervals (CI) among SSA residents and residents in the diaspora. The adjusted coefficients (β) with their 95% confidence intervals obtained from the multiple linear regression model were used to measure the factors associated with the risk perception of COVID-19 among SSA residents and those in the diaspora. Only significant variables in the univariate analysis were used to build the regression model. Knowledge was included in the model because it is strongly related to attitude and practice, while knowledge and attitude have been reported to be associated with practice ([30]). Feeling about the practice of self-isolation during the COVID-19 lockdown would help in identifying individuals who could develop mental health issue during the lockdown because past studies showed that longer duration of separation and restriction of people’s movement due to SARS were associated with poorer mental health [31, 32]. Including attitude towards the mitigation practices in the model would influence action to reduce the spread of the infection. In our linear regression analyses, we checked for homogeneity of variance and multicollinearity, including Variance Inflation Factors (VIF) and the VIF < 4 was considered suitable [33]. All analysis was performed using Stata version 14.1 (StataCorp 2015. College Station, United States of America), and a two-tailed *p*-value < 0.05 was considered statistically significant.

## Results

### Demographics of respondents in Africa and in the diaspora

**Fig. 1:**
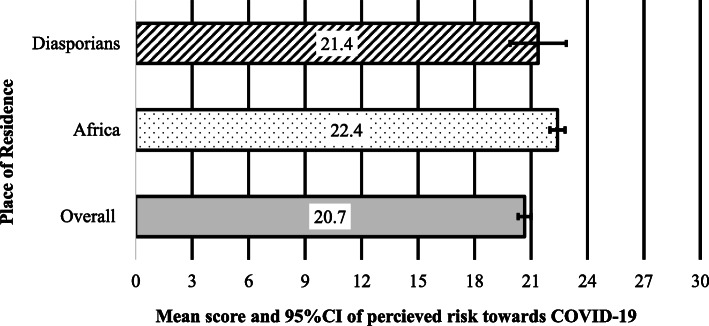
Mean scores (/30) of risk perception towards COVID-19 among Sub-Saharan Africans living locally (Africa) and in the diaspora. *Error bars are 95% confidence intervals of mean scores*

Of the 1969 respondents (55.1% male and 44.9% female) that completed the survey, the majority were living in SSA (*n* = 1855, 92.8%) and 143 (7.2%) in the diaspora. The percentage distribution of the respondents by country of residence for local residents and those in diaspora has been presented as a Supplementary figure. The majority of the local respondents lived in Ghana (28.2%), followed by Nigeria (26.7%) and South Africa (21.7%), while many of those in diaspora were from the USA (19.6%), then UK (18.2%) and Australia (15.4%). Figure 1 presents the mean scores (out of 30) and the 95% CI of risk perception scores towards COVID-19 based on respondents region of residence. There was no significant difference in the mean risk perception scores between the two groups (*p* = 0.117). Table 2 shows the demographics of SSA in Africa and in the diaspora with their mean (standard deviation) scores for perceived risk towards COVID-19. Compared to residents in Africa, those living in the diaspora were younger, more often female, and less often married.

**Table 2:** Demographics of Sub-Saharan Africans living in Africa and in the diaspora with their mean (standard deviation) scores for the perceived risk of contracting COVID-19

Variables	Local SSA	Scores	*P*-value	Diaspora SSA	Scores	*P*-value
Demography						
**Age category in years,** ***n*** **= 1818(**^b^**)**						
18–28	722	20.0 (8.1)	**0.003**	52	20.7 (8.1)	0.371
29–38	476	21.3 (7.3)		47	20.2 (7.5)	
39–48	393	21.3 (7.7)		31	18.3 (8.9)	
**49+**	227	21.6 (7.1)		13	22.5 (5.6)	
**Sex,** ***n*** **= 1822**						
Males	1002	21.0 (7.6)	0.394	80	21.0 (7.0)	0.118
Females	820	20.7 (7.9)		62	18.9 (8.8)	
**Marital status,** ***n*** **= 1825**						
Married	793	21.1 (7.4)	0.293	70	20.1 (8.2)	0.929
Not married^a^	1032	20.7 (8.0)		73	20.2 (7.7)	
**Education status,** ***n*** **= 1827 (**^b^**)**						
Postgraduate education (Masters /PhD)	576	21.3 (6.8)	**< 0.001**	56	20.4 (7.7)	0.918
Bachelor education	861	21.1 (7.8)		64	20.1 (8.2)	
Secondary/Primary education	390	19.1 (9.0)		23	19.5 (7.2)	
**Employment status,** ***n*** **= 1830**						
Employed	1200	21.1 (7.5)	**0.040**	97	19.8 (7.7)	0.462
Not employed	630	20.3 (8.2)		46	20.9 (8.3)	
**Religion,** ***n*** **= 1825**						
Christianity	1605	20.8 (7.7)	0.510	136	20.2 (7.8)	0.802
Others	220	21.2 (7.6)		7	19.4 (9.6)	
**Occupation, 1753**						
Non-health care sector	1357	20.6 (7.8)	0.109	111	19.6 (8.1)	0.743
Health care sector	396	21.3 (7.8)		34	20.2 (8.9)	
**Household factors**						
Do you live alone during COVID-19, *n* = 1826						
No	1483	20.8 (7.6)	0.864	117	20.0 (7.8)	0.860
Yes	343	20.9 (8.1)		26	20.3 (8.6)	
Number living together, *n* = 1650 (^b^)						
1–3 people	466	20.9 (7.5)	0.866	36	18.9 (8.9)	0.249
4–6 people	870	20.7 (7.9)		37	17.5 (10.2)	
6+ people	314	21.0 (7.7)		26	21.3 (6.4)	
Public Attitude towards mitigation measures						
**Practiced self-isolation**, *n* = 1644						
No	1141	22.8 (4.7)	0.390	83	21.9 (5.3)	0.871
Yes	503	23.0 (5.1)		50	21.8 (5.7)	
**Practiced home quarantine,** ***n*** **= 1641**						
No	989	22.8 (4.7)	0.814	91	21.7 (5.3)	0.496
Yes	652	22.9 (4.9)		42	22.4 (5.9)	
**Feeling about the self-isolation**						
*Anxious, n = 1463*						
No	592	20.8 (7.7)	0.865	50	21.0 (6.8)	0.213
Yes	871	20.7 (8.1)		62	19.0 (9.4)	
*Bored, n = 1493*						
No	444	20.7 (7.9)	0.990	30	19.9 (8.1)	0.897
Yes	1049	20.7 (7.9)		87	20.1 (8.3)	
*Frustrated, n = 1467*						
No	704	20.7 (7.8)	0.982	63	20.5 (8.4)	0.657
Yes	763	20.7 (8.2)		56	18.4 (8.2)	
*Angry, n = 1418*						
No	1098	20.8 (8.0)	0.692	88	22.4 (9.5)	0.283
Yes	320	20.6 (7.8)		23	19.7 (9.2)	
**Knowledge scores**^c^	1855	7.2 (2.2)		150	7.2 (2.5)	
**Attitude scores**	1855	13.7 (5.2)		150	14.0 (5.5)	

*Abbreviation: COVID-*19 Coronavirus diseases 2019

For each variable, no of responses = 1969 otherwise indicated

*P*-values are results of independent t-test and analysis of variance

^a^single, divorced and widowed

^b^Analysis of variance (ANOVA) was used

^c^continuous variables

### Perception of overall COVID-19-associated risk

For those in SSA, the risk perception score was significantly lower in the 18–28 years age group (*p* = 0.003, Table 2) than in older age groups. Again, employment (*p* = 0.040) and a higher level of education (*p* < 0.001, Table 2) were significantly associated with higher risk perception scores than being unemployed and having a lower education, respectively. There was no significant difference in the risk perception scores based on gender, marital status, religion, occupation, and the number of people living together among SSA residents. The risk perception score did not significantly differ across the sociodemographic characteristics of the participants living in the diaspora.

Among those living in SSA and those in the diaspora, the mean scores for risk perception was similar between those who either practiced or did not practice self-isolation and home quarantine. Similarly, no significant differences in risk perception were observed between participants who reported being anxious, bored, frustrated, angry compared to those who did not report any of these symptoms in the two groups.

Table 3 shows the unadjusted and adjusted coefficients for factors associated with risk perception of COVID-19 among Africans residing in SSA. In contrast, Table 4 shows the same information for those living in the diaspora. Among the local SSA residents, working in the health care sector (adjusted coefficient, β = 0.61, 95% CI [0.09, 1.14]) was associated with high-risk perception towards COVID-19, as well as knowledge (β = 1.19, 95% CI [1.05, 1.34]) and attitude (β = 0.63, 95% CI [0.58, 0.69]) towards COVID-19 mitigation measures (Table 3). Although, unemployment (B = − 0.78, 95% CI [− 5.53, − 0.04]) and lower levels of education (primary/secondary education, B = − 2.19, 95% CI [− 3.32, − 1.05]) were significantly associated with lower risk perception towards COVID-19 in the univariate analysis, the significance was lost after adjusting for other potential confounding factors.

**Table 3:** Unadjusted and adjusted coefficients for factors associated with perceived risk of contracting Coronavirus diseases (COVID-19) among SSAs living in African countries

Variables	Unadjusted Coefficient	95%CI	Adjusted Coefficient	95%CI
Demography				
**Age category in years**				
18–28	0.00		0.00	
29–38	**1.29**	**0.40, 2.18**	0.49	−0.06, 1.05
39–48	**1.30**	**0.35, 2.24**	**0.60**	**0.01, 1.19**
49+	1.59	0.44, 2.73	0.29	−0.43, 1.01
**Sex**				
Males	0.00		–	–
Females	−0.31	−1.02, 0.40		
**Marital status**				
Married	0.00		–	–
Not married	−0.38	−1.10, 0.33		
**Education status**				
Postgraduate education (Masters /PhD)	0.00		–	–
Bachelor education	− 0.20	− 0.98, 0.59		
Secondary/Primary education	**−2.19**	**−3.32, −1.05**		
**Employment status**				
Employed	0.00		–	–
Not employed	**−0.78**	**− 1.53, − 0.04**		
**Religion**				
Christianity	0.00		–	–
Others	0.37	−0.72, 1.45		
**Occupation**			–	–
Non-health care sector	0.00		0.00	
**Health care sector**	0.71	−0.16, 1.59	**0.61**	**0.09, 1.14**
**Household factors**				
Do you live alone during COVID-19				
No	0.00		–	–
Yes	0.08	−0.83, 0.99		
Number living together				
< 3 people	0.00		–	–
4–6 people	−0.17	−1.05, 0.70		
6+ people	0.07	−1.04, 1.18		
Public Attitude towards COVID-19 Mitigation measures				
**Practiced self-isolation**				
No	0.00		–	–
Yes	0.22	−0.28, 0.72		
**Practiced home quarantine**				
No	0.00		–	–
Yes	0.06	−0.42, 0.53		
**Feeling about the self-isolation**				
*Anxious*				
No	0.00		–	–
Yes	−0.07	−0.90, 0.76		
*Bored*				
No	0.00		–	–
Yes	0.01	−0.87, 0.88		
*Frustrated*				
No	0.00		–	–
Yes	−0.01	−0.83, 0.81		
*Angry*				
No	0.00		–	–
Yes	−0.20	−1.19, 0.79		
**Knowledge score**^a^	**2.38**	**2.26, 2.50**	**1.19**	**1.05, 1.34**
**Attitude score**^a^	**1.08**	**1.08, 1.13**	**0.63**	**0.58, 0.69**

COVID-19 Coronavirus diseases 2019

^a^continuous variables

Confidence intervals (CIs) not including 0 are significant variables

**Table 4:** Unadjusted and adjusted coefficients and 95% confidence intervals (CI) of factors associated with perceived risk of contracting Coronavirus diseases (COVID-19) among SSAs living in the diaspora

Variables	Unadjusted Coefficient	95% CI	Adjusted Coefficient	95% CI
Demography				
**Age category in years**				
18–28	0.00		–	–
29–38	−0.54	−3.68, 2.60		
39–48	−2.45	−6.00, 1.09		
49+	1.75	−3.09, 6.59		
**Sex**				
Males	0.00		–	–
Females	−2.08	−4.70, 0.53		
**Marital status**				
Married	0.00		–	–
Not married	0.12	−2.50, 2.74		
**Education status**				
Postgraduate Degree (Masters /PhD)	0.00		–	–
Bachelor’s degree	−0.35	−3.13, 2.44		
Secondary/Primary	−0.97	−5.81, 3.87		
**Employment status**				
Employed	0.00		–	–
Unemployed	1.04	−1.76, 3.84		
**Religion**				
Christianity	0.00		–	–
Others	−0.77	−6.84, 5.30		
**Occupation**				
Non-health care sector	0.00		–	–
Health care sector	0.53	−2.68, 3.75		
**Household factors**				
Do you live alone during COVID-19				
No	0.00		–	–
Yes	0.30	−3.09, 3.70		
Number living together				
< 3 people	0.00		–	–
4–6 people	−1.43	−5.55, 2.70		
6+ people	2.38	−2.15, 6.92		
Public Attitude towards COVID-19 mitigation measures				
**Self-isolation**				
No	0.00		–	–
Yes	−0.16	−2.10, 1.78		
**Home quarantined**				
No	0.00		–	–
Yes	0.70	−1.32, 2.72		
**Feeling about the self-isolation**				
*Anxious*				
No	0.00		–	–
Yes	−1.98	−5.13, 1.16		
*Bored*				
No	0.00		–	–
Yes	0.23	−3.22, 3.67		
*Frustrated*				
No	0.00		–	–
Yes	−0.67	−3.65, 2.31		
*Angry*				
No	0.00		–	–
Yes	−2.11	−5.98, 1.77	0.00	
**Knowledge score**^a^	**2.36**	**1.97, 2.75**	**1.79**	**1.16, 2.41**
**Attitude score**^a^	**0.99**	**0.81, 1.17**	**0.30**	**0.02, 0.58**

COVID-19 Coronavirus diseases 2019

^a^continuous variables

Confidence intervals (CIs) not including 0 are significant variables

From Table 4, it can be seen that, among SSAs in the diaspora, knowledge (β = 1.79, 95% CI [1.16, 2.41]) and attitude (β = 0.30, 95% CI [0.02, 0.58]) were similarly associated with a high-risk perception of COVID-19. However, there was no significant association between the demographic variables and the risk perception scores in this group.

## Discussion

This study found comparable high-risk perception scores among residents living in SSA and those in the diaspora, which were associated with an increase in knowledge of COVID-19 and attitude towards the mitigation measures. Health care workers resident in SSA had higher risk perception scores compared to their counterpart non-healthcare workers. Although having lower education and not working during the pandemic were associated with lower risk perception of COVID-19 among local residents, this association was nullified after adjusting for other demographic variables.

The finding that older individuals felt at greater risk of COVID-19 was in line with past studies showing that older individuals have significantly higher COVID-19 related severe complications and deaths than young individuals [34]. Public awareness of this information may explain the finding of lower risk perception for contracting the infection among younger respondents in SSA. As highlighted by Dillard et al. [35], having a perceived low risk of infection can make young people become less compliant to public health measures. This can in turn lead to higher COVID-19 infection [35], and ultimately passing the infection to the population more susceptible to COVID-19 related complications since young people were shown to be more likely to transmit the virus than others [36]. In line with these findings, some countries took stringent steps to limit the young population from transmitting COVID-19 infection to the older population [37–40] but recorded mixed success [40–42]. Rapid and proactive outreach programs targeted at young people in Australia and Canada might explain why the risk perception was similar between younger and older participants living in the diaspora in this study [43]. Such directed programs and policies should be implemented within the vulnerable groups in our local populations.

Studies have reported a high perceived risk of COVID-19 among African health care workers [44–46] but did not compare between health and non-health care workers. In a cross-sectional study conducted on 350 Ghanaians during the early stage of the COVID-19 outbreak, there was no difference in risk perception scores between healthcare and non-healthcare workers [47] but healthcare workers reported higher mean scores than non-healthcare workers. The higher mean score recorded among healthcare worker in this study may be attributed to the fact that healthcare workers had to work even if their individual risk perception would want to make them to comply with risk mitigation measures such as isolation [46, 48]. In this study, high-risk perception for contracting COVID-19 was associated with working in the health care sector, but this was only significant in the SSA residents. Firsthand experience with the virus is often linked to high-risk perception [49], and higher knowledge of the disease among health care workers than the non-health care workers might explain their higher perception of risk for contracting the infection. The lack of proper training on protective measures reported in previous studies by health workers in SSA countries [46] may explain the significant association found among local health care workers but not among those living in the diaspora. Again, the implementation of targeted policies may as well account for the lack of association among respondents living abroad.

In this study, knowledge about COVID-19 and a positive attitude towards the mitigation measures were associated with a high-risk perception of contracting the disease, both in SSA and in the diaspora. Similar findings have been reported in Ethiopia [50], where individuals who perceived a higher risk were more likely to adopt protective measures, which in turn influences the probability of infection [50, 51]. However, the prevalence of misinformation about COVID-19 among SSA respondents [52], together with the psychological stress caused by themisinformation about COVID-19 due to the poor knowledge about the disease [28], are potential sources of reduced risk perception in this sub region. These would lead to increased transmissions and mortality. Hence, accurate information about the pandemic using the trusted media platforms can help in proper risk judgement and adoption of public health measures to control the spread of infection [28, 53].

COVID-19 related morbidity and mortality vary disproportionately based on sociodemographic characteristics, for instance, males and older people have high mortality due to COVID-19 compared to females and the young population [54]. Individuals' behaviours towards safety measures have been linked to their level of the perceived risk of disease [35]. Adopting public health measures such as the use of a nose mask in public areas and frequent hand sanitization can lead to successful control of air-borne infectious diseases like COVID-19 [53]. Therefore, public health strategies for successful control of COVID-19 among SSAs may be beneficial by targeting the sub-population identified in this study. This includes, the unemployed, non-healthcare workers, the younger population and those with lower education.

This study was limited by several factors: 1), the assessed risk perception and comparison of the perception scores from SSA residents in and outside Africa may be limited by the fact that those who felt they were at risk of COVID-19 infection were more likely to respond to recommended health behaviours [55]; 2), findings from this study cannot be generalized to the entire SSA regions; 3), it was an online survey made available only in English language thus restricting respondents without access to the internet or where internet penetration remains relatively low and some from French-speaking SSA nations [56]; 4), the survey items were self-administered and some of the questions for example, those on compliance require subjective responses, and has no answer that can be verified. If a respondent reported good behaviour but did not practice it, there is no way we can independently verify their responses. Despite these limitations, this study from SSA region provided insight into the role of residence in mitigating the factors that influence risk perception of COVID-19 among SSAs during the pandemic. The study used a robust analysis to control for potential confounders during the analysis in order to reduce the issue of bias.

## Conclusions

In summary, this study explored the factors associated with the risk perception of contracting COVID-19 among SSAs, particularly looking at the role of residence in peoples’ level of risk perception. The findings indicate that health communication and education strategies designed to promote the adoption of preventive behaviours among SSAs should focus on increasing knowledge about the disease and encouraging a positive attitude towards the mitigation measures. In addition, such programmes will benefit from targeting the unemployed, less educated, healthcare workers and the younger population, for optimum outcome. These findings can be helpful in policy implications in disaster risk management, including the control of COVID-19, particularly in English speaking countries in the SSA region.

## Supplementary Information

**Additional file 1: Supplementary Figure**. Percentage distribution of respondents by country of residence for local and diaspora residents.

**Additional file 2: Supplementary Table**. Sample of Survey items with response options.

## Data Availability

The datasets used and/or analysed during the current study are available from the corresponding author on reasonable request.

## Abbreviations

COVID-19: *Coronavirus* SARS-CoV2
SSA: Sub-Sahara Africa
CI: Confidence intervals
EVD: Ebola Virus Disease
EPPM: Extended parallel process model
WHO: World Health Organization
SPRP: Strategic Preparedness and Response Plans
ANOVA: Analysis of variance
