# Supplementary figures and images for: Risk perception of COVID-19 among sub-Sahara Africans: a web-based comparative survey of local and diaspora residents
Source: BMC Public Health. 2021 Aug 18;21:1562. doi: 10.1186/s12889-021-11600-3 (PMC8370831; doi:10.1186/s12889-021-11600-3)

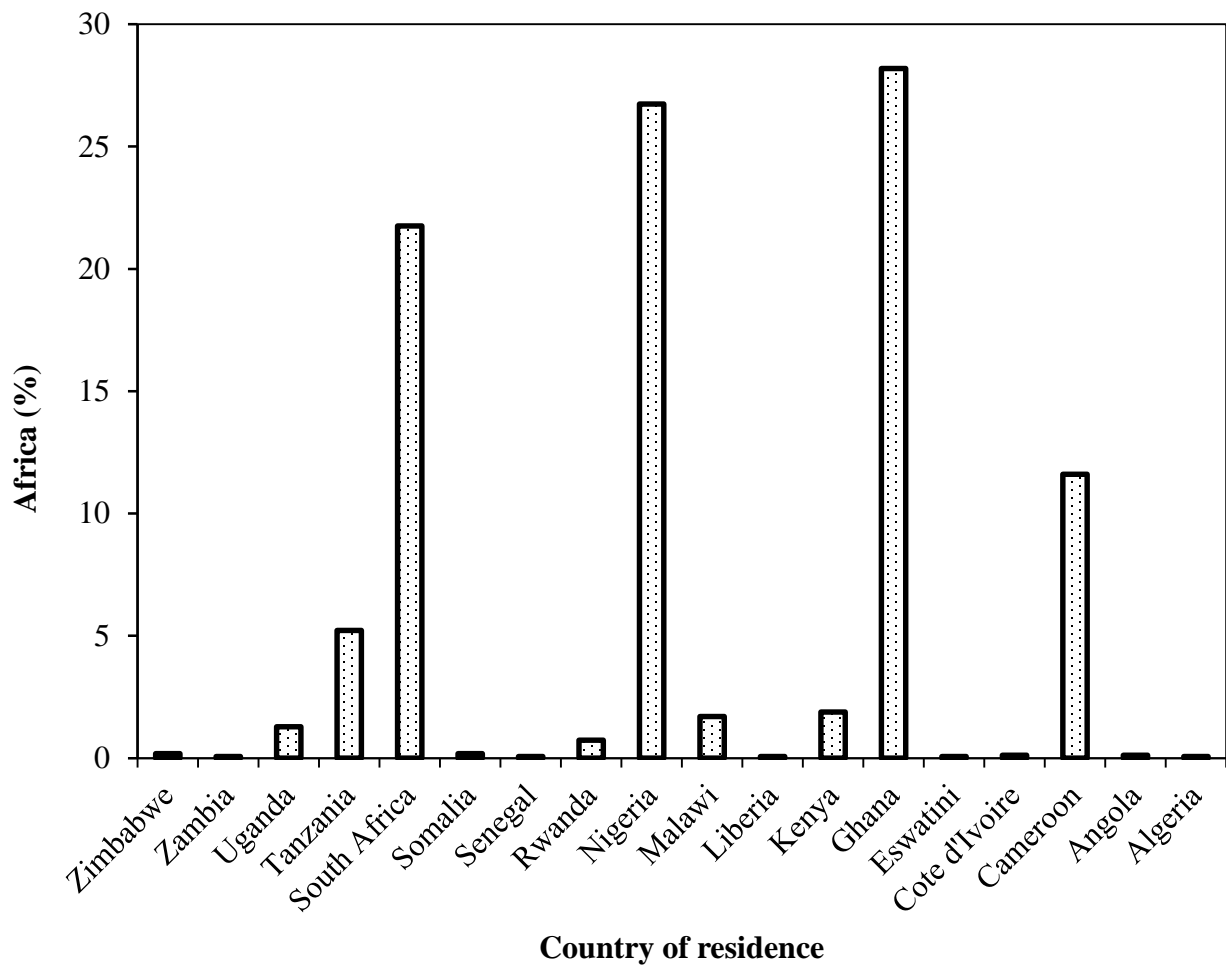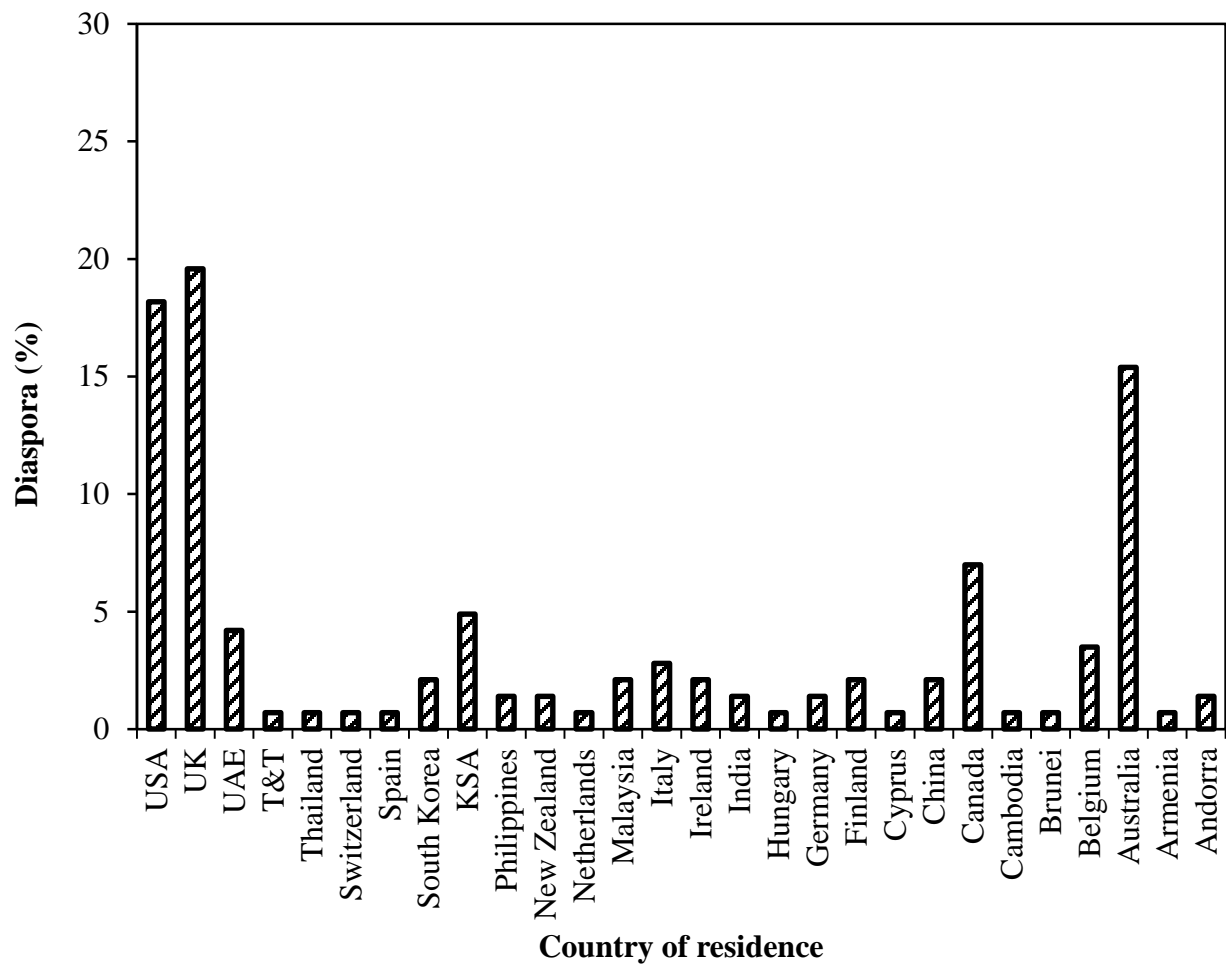

Supplement: Supplementary file 1 — Additional file 1: Supplementary Figure. Percentage distribution of respondents by country of residence for local and diaspora residents. [file 12889_2021_11600_MOESM1_ESM.pdf]
