# Supplementary material for: Risk perception of COVID-19 among sub-Sahara Africans: a web-based comparative survey of local and diaspora residents
Source: BMC Public Health. 2021 Aug 18;21:1562. doi: 10.1186/s12889-021-11600-3 (PMC8370831; doi:10.1186/s12889-021-11600-3)
Supplement: Supplementary file 2 — Additional file 2: Supplementary Table. Sample of Survey items with response options. [file 12889_2021_11600_MOESM2_ESM.docx]

**Supplementary Table 1:** Sample of Survey items with response options**.**

Knowledge about COVID-19 infection

K1. Are you aware of the Coronavirus disease (COVID-19) outbreak? [YES/NO]

K2. Are you aware of the origin of the Coronavirus disease (COVID-19) outbreak? [YES/NO]

K3. Do you think Coronavirus disease (C0VID-19) outbreak is dangerous? [YES/NO]

K4. Do you think Hand Hygiene / Hand cleaning is important in the control of the spread of the Coronavirus disease (COVID-19) outbreak? [YES/NO/NOT SURE]

K5. Do you think ordinary residents can wear general medical masks to prevent the infection by the COVID-19 virus? [YES/NO/NOT SURE]

K6. Do you think there are any specific medicines to treat Coronavirus disease (COVID-19)?

[YES/NO/NOT SURE]

K7. The main clinical symptoms of Coronavirus disease (COVID-19) are: (Type "YES" or "NO" to the suggested options as applicable)

- Fever
- Fatigue
- Dry cough
- Sore throat

K8. Unlike the common cold, stuffy nose, runny nose, and sneezing are less common in persons infected with the COVID-19 virus. [TRUE/FALSE/NOT SURE]

K9. There currently is no effective cure for COVID-2019, but early symptomatic and supportive treatment can help most patients recover from the infection [TRUE/FALSE/NOT SURE]

K10. It is not necessary for children and young adults to take measures to prevent the infection by the COVID-19 virus. [TRUE/FALSE/NOT SURE]

K11. COVID-19 individuals cannot spread the virus to anyone if there is no fever [TRUE/FALSE/NOT SURE]

K12. The COVID-19 virus spreads via respiratory droplets of infected individuals. [TRUE/FALSE/NOT SURE]

K13. To prevent being infected by Coronavirus disease (COVID-19), individuals should avoid going to crowded places such as train stations, religious gatherings, and avoid taking public transportation. [TRUE/FALSE/NOT SURE]

K14. Isolation and treatment of people who are infected with the Coronavirus disease (COVID-19) virus are effective ways to reduce the spread of the virus. The observation period is usually 14 days. [TRUE/FALSE/NOT SURE]

K15. Not all persons with COVID-2019 will develop to severe cases. Only those who are elderly, have chronic illnesses, and are obese are more likely to be severe cases. [TRUE/FALSE/NOT SURE]

Risk Perception

Please rate your chances of personal risk of infection with COVID-19 for each of the following?

P1. Risk of becoming infected.[VERYHIGH/HIGH/LOW/VERY LOW/UNLIKELY]

P2. Risk of becoming severely infected. [VERY HIGH/HIGH/LOW/VERY LOW/UNLIKELY]

P3. Risk of dying from the infection [VERY HIGH/HIGH/LOW/VERY LOW/UNLIKELY]

P4. How much worried are you because of COVID-19? [A GREAT DEAL/A LOT/ A MODERATE AMOUNT/A LITTLE/NOT ALL]

P5. How likely do you think Coronavirus disease (COVID-19) will continue in your country?[VERY HIGH/HIGH/LOW/VERY LOW/UNLIKELY]

P6. If Coronavirus disease (COVID-19) continues in your country, how concerned would you be that you or your family would be directly affected? [EXTREMELY CONCERNED/CONCERNED/NEITHER CONCERNED, NOR UNCONCERNED/UNCONCERNED/EXTREMELY UNCONCERNED

P7-P11. How do you feel about the Self-isolation? (Type "YES" or "NO" to the suggested options as applicable)

- - Worried
  - Bored
  - Frustrated
  - Angry
  - Anxious

P12. Are you angry because of the quarantine measures? [YES/NO]

Attitude towards public health practices to mitigate the spread of COVID-19 (Compliance)

A1. Are you currently or have you been in (domestic/home) quarantine because of COVID-19? [YES/NO]

A1. Are you currently or have you been in self-isolation because of COVID-19? [YES/NO]

A3. In recent days, have you gone to any crowded place including religious events? [ALWAYS/SOMETIMES/RARELY/NOT AT ALL/NOT SURE]

A4. In recent days, have you worn a mask when leaving home? [ALWAYS/SOMETIMES/RARELY/NOT AT ALL/NOT SURE]

A5. In recent days, have you been washing your hands with soap and running water for at least 20 seconds each time?[ALWAYS/SOMETIMES/RARELY/NOT AT ALL/NOT SURE]

A6. Since the government gave the directives on preventing getting infected, have you procured your mask and possibly sanitizer?[YES/NO]

A7. Have you travelled outside your home in recent days using the public transport [YES/NO]

A8. Are you encouraging others that you come in contact with to observe the basic prevention strategies suggested by the authorities?[YES/NO]
